# Diseases of the Fingers

**Published:** 1891-09-05

**Authors:** 


### SURGERY.

### DISEASES OF THE FINGERS.

#### E.—Diseases of the Tendons.

Besides those already mentioned under the heading "Contractions," &c., there are two conditions especially worthy of notice, viz., "Tenosynovitis" and "Ganglion." Both are common affections, and cause great impairment of the use of the fingers, although not actually situated on them usually.

1. *Tenosynovitis or Thecitis.*—Inflammation of the tendons and their sheaths occurs as a secondary affection in caries, necrosis, &c., and as a result of whitlow; it is also found in gout and rheumatism. The commonest form, however, is that which follows sprains and strains of the wrist and overwork of the extensor and flexor muscles. The symptoms are pain of a dull, aching character and loss of power in the affected tendon. It is not restricted to any period of life, but is most frequent in the young. Occupations requiring activity in one set of muscles are exciting causes. There is a dry, fine crepitation to be felt when the tendon is used which is quite diagnostic. Besides this there is sometimes a distinct "catch" on passive movement.

Amongst the labouring classes the extensors of the thumb, especially the extensor ossis metacarpi pollicis and the extensor primi internodii, are, perhaps, the most usual seats of the disease. The result is an elongated, spindle-shaped swelling reaching from the base of the thumb across the back of the wrist and fore-arm. On grasping this firmly and then moving the thumb, the peculiar grating sensation is at once apparent, and is obviously due to roughening of the synovial sheath. The treatment consists in complete rest, and in order to procure this it is necessary to bandage the hand and fore-arm on a splint which is broad enough to support the thumb. This gives much better results than the ordinary method of leaving the thumb only half confined. At the end of a week or so, passive movement and rubbing should be employed. As a rule, patients will not carry out the latter unless they have something to "rub in." Hence the practice of giving soap liniment or camphorated oil " to be used night and morning."

If the affection does not get well under this treatment, it is best to try counter-irritation with iodine or "Scott's dressing," and in some cases it is necessary to open the sheath and let out the fluid. This should not be done until a fair trial has been given of other methods. If very obstinate, the general health should be looked to, and any gouty or rheumatic tendency treated by appropriate medicines. When the disease has become chronic (which results usually from the patient persisting in using the hand before the first effusion is absorbed), an incision may be made, and the tendon-sheath scraped out. Gelatinous fluid is often found, and sometimes the remarkable "melon-seed" bodies, which are looked upon by one German surgeon as characteristic of tubercle. However this may be, their removal is essential. In the experience of the writer they are mostly found in tenosynovitis of the flexor tendons, and more in the fingers and palm than in the wrist.

2. *Ganglion.*—These small swellings are found on any of the tendons of the wrist or fingers, but are most common on the common extensor. The symptoms are pain, sometimes very severe, when the muscle is used, and occasionally great loss of power in the movements of the hand, resembling, as before stated, "writer's cramp." In an article in the *Medical Journal* of April 25, 1891, Mr. Walter Spencer comes to the conclusion that "ganglion" begins either as tenosynovitis, which becomes localised in one spot, or from a sprain or blow causing an immediate effusion of blood into the tendon sheath. His idea is that the initial inflammation shuts off a portion of this sheath, which constitutes the round or oval swelling. If this is the case the tendon would form part of the wall of the tumour, and the two would move together. In the majority of ganglia it is impossible to work the tendon without moving the swelling, but this is not invariably the case, and there is not sufficient evidence to show that this affection is not caused, as other surgeons believe, by a small rupture (from over-strain or blow, &c.) of the sheath into the surrounding cellular tissue. This would form a small cyst, which would soon acquire a distinct wall. Probably both views are correct, and we may assume that there are three ways in which the disease may originate, viz. (1) by rupture of the sheath and escape of fluid into the surrounding tissues; (2) by the partial rupture and slow dilatation of a portion of the sheath; and (3) as indicated by Mr. Spencer. The treatment depends on the thickness of the ganglion wall, and this is not always easy to ascertain. If there is distinct fluctuation (and it is probably thin walled) a fine trocar should be introduced, the contents evacuated, and firm pressure kept up for two or three days. The injection of iodine should not be practised, for in those cases where the tumour surrounds a tendon, enough inflammation may be excited by the irritating fluid to bind the tendon firmly to the adjacent parts, a result of course to be deprecated. Should the swelling reappear, it is better to cut it open with antiseptic precautions and insert a small horsehair drain. This will cause granulations to spring up, and the cavity will permanently close. It may be mentioned that in the early stages counter-irritation and strapping will sometimes effect a cure. In chronic thick-walled ganglia it is often necessary to excise, great care being taken to remove only the part furthest from the tendon if the two are intimately joined.

*Bursitis* over the [illegible]oints of the knuckles is a rare disease, except in gouty and rheumatic subjects. There is generally a preceding thickening of the fibrous tissue round the joint, probably with concretions of urates. The bursa formed is in the subcutaneous areolar tissue. General treatment is necessary in most cases, and the swelling is often intractable. Iodine, iodide of potassium ointment and blisters may be used locally, and salicylate of sodium or lithium should be given as medicines. Puncture and evacuation of the contents, perhaps with scraping, may be necessary, but should not be employed until other measures have had a prolonged and patient trial.

